# 2-Aminoethyl Dihydrogen Phosphate (2-AEH2P) Associated with Cell Metabolism-Modulating Drugs Presents a Synergistic and Pro-Apoptotic Effect in an In Vitro Model of the Ascitic Ehrlich Tumor

**DOI:** 10.3390/biomedicines12010109

**Published:** 2024-01-04

**Authors:** Monique G. Alves, Laertty G. S. Cabral, Paulo G. F. Totti, Felipe R. Azarias, Karine T. Pomini, Rose E. G. Rici, Rosa A. N. Laiso, Durvanei A. Maria

**Affiliations:** 1 Development and Innovation Laboratory, Butantan Institute, São Paulo 05359-900, Brazil; laertty.c@usp.br (L.G.S.C.); felipe.azarias@outlook.com (F.R.A.);; 2 Graduate Program in Medical Sciences, College of Medicine, University of São Paulo, São Paulo 05508-220, Brazil; 3 Graduate Program in Structural and Functional Interactions in Rehabilitation, Postgraduate Department, University of Marília (UNIMAR), Marília 17525-902, Brazilroseeli@usp.br (R.E.G.R.); 4 Graduate Program in Anatomy of Domestic and Wild Animals, College of Veterinary Medicine and Animal Science, University of São Paulo, São Paulo 05508-220, Brazil

**Keywords:** breast cancer, 2-AEH2P, phospholipid, apoptosis, metabolism modulators, tumor microenvironment

## Abstract

The progression and maintenance of cancer characteristics are associated with cellular components linked to the tumor and non-cellular components with pro-tumoral properties. Pharmacological association with antagonists of the cellular components of the tumor, such as anti- and pro-apoptotic drugs, represents a novel adjuvant strategy. In this study, the antiproliferative, pro-apoptotic, and pharmacological effects of the combination of monophosphoester 2-AEH2P with Simvastatin, Coenzyme Q10, the chemotherapeutic drug paclitaxel, and colony-stimulating factor (GM-CSF) were evaluated. Tests were conducted to determine cytotoxic activity using the MTT method, cell cycle phases, and fragmented DNA by flow cytometry, mitochondrial membrane potential, expression of cell markers Bcl2, TNF-α/DR-4, Cytochrome c, caspase 3, and P53, and analysis of drug combination profiles using Synergy Finder 2.0 Software. The results showed a synergistic effect among the combinations, compared to individual treatments with the monophosphoester and other drugs. In addition, there was modulation of marker expression, indicating a pro-apoptotic and immunomodulatory effect of 2-AEH2P. Pharmacological analysis revealed that tumor cells treated with GM-CSF + 2-AEH2P exhibited a synergistic effect, while groups of tumor cells treated with paclitaxel, Coenzyme Q10, and Simvastatin showed additive effects. Furthermore, treatment with the paclitaxel + 2-AEH2P combination (12 h) resulted in a significant reduction in mitochondrial membrane potential. Pharmacological combinations for normal cells did not exhibit deleterious effects compared to mammary carcinomatosis tumor (EAT) cells.

## 1. Introduction

Considering that the acquisition and maintenance of the characteristics of tumor cells depend on contributions from the tumor microenvironment (TME), restricting non-tumor host cells or non-cellular components with pro-tumoral functions may be an effective strategy for the development of new therapies [1]. Tumor heterogeneity is the main cause of drug resistance, as it directly affects therapeutic targets, constantly reprogramming the tumor microenvironment (TME) [2]. In ER+ breast cancer, responsible for approximately 75% of all breast cancers, resistance to endocrine therapies poses a significant challenge, with alterations in the TME being one of these factors [3]. Similarly, triple-negative breast cancer cells (TNBC) still lack specific treatment strategies and are treated with conventional therapy, often leading to systemic recurrence [4]. The growing understanding of the importance of the TME in cancer biology has brought about a shift from a cancer-centric model to a model that considers the TME as a whole [5]. Currently, studies classify the TME into six specialized microenvironments: hypoxic niche, immunological microenvironment, metabolic microenvironment, acidic niche, innervated niche, and mechanical microenvironment [6]. Within this context, considering the critical role of lipids in cancer progression, targeting the lipid metabolism pathway may provide a new therapeutic approach [7]. In addition, beyond its primary role as a structural component of the membrane matrix, altered lipid metabolism also presents important secondary messengers and serves as a source for energy production [8]. It has been reported that cholesterol synthesis is crucial for the formation and invasion of breast cancer cells. Furthermore, its positive regulation is associated with the promotion of oncogenic processes such as initiation, migration, and tumor angiogenesis, and influences the immune landscape [9,10]. Moreover, a Gene Ontology analysis identified that genes positively regulated in the mevalonate pathway (MVA), 3-β-hydroxy-3-β-methylglutaryl coenzyme A synthase 1 (HMGCS1) and 3-β-hydroxy-3-β-methylglutaryl coenzyme A reductase, are related to acquired resistance to doxorubicin in in vitro models [11]. Supporting this hypothesis, Wang et al. significantly reduced the resistance of in vitro and in vivo tumor cells to platinum-derived chemotherapy by lowering cholesterol levels [12]. In a previous study involving human breast cancer cell lines MDA-MB-231, MDA-MB-468, MCF-7, and T47D, differences in individual sensitivity to statins, inhibitors of 3-hydroxy-3-methylglutaryl-CoA, were identified, indicating resistance of tumor cells to statins [13]. Therapeutic strategies related to the immune microenvironment are also evaluated for their effectiveness in combating breast cancer. Granulocyte-macrophage colony-stimulating factor (GM-CSF) has been employed as an adjuvant in cancer immunotherapy [14]. HER2/neu peptide (GP2)-based breast cancer vaccines with GM-CSF as an adjuvant have shown promising results, with the GP2+GM-CSF vaccine combination capable of eliciting both ex vivo and in vivo immune responses [15,16,17,18]. Conversely, a study investigating the adjuvant properties of IFN-γ and GM-CSF in a vaccine against tumors expressing carcinoembryonic antigen (CEA) found no additional effect on tumor protection when GM-CSF was added [19]. As for Coenzyme Q10, contradictions arise regarding its therapeutic use. Under hypoxic conditions, Coenzyme Q10 suppressed the expression of HIF-1α, potentially contributing to the inhibition of NLRP3-mediated inflammation, metastasis, and effects on the Warburg metabolism in MDA-MB-231 cells [20]. However, the FDA does not approve its use to treat any medical condition, and its consumption is widely available only as a dietary supplement. It is essential to note that such supplements are not formally analyzed for manufacturing consistency and may vary considerably from batch to batch [21]. The FDA’s verdict is further substantiated by a recent observational study suggesting that the use of antioxidant supplements, such as Coenzyme Q10, may be associated with increased recurrence rates and decreased survival after evaluating 1134 breast cancer patients [22]. The NCI’s PDQ Cancer Information Summary on cancer prevention succinctly outlines the risks of Coenzyme Q10 use, emphasizing its use with other conventional therapies, including chemotherapy, radiotherapy, and surgery, indicating limited investigation as a cancer treatment in humans [23]. Therefore, it is still necessary to investigate whether, when associated with another potential drug with antitumoral properties, Coenzyme Q10, as a modulating adjuvant, will exhibit beneficial results. With this aim, our group has expressed interest in exploring the activity of 2-aminoethyl dihydrogen phosphate (2-AEH2P) as a therapeutic adjuvant through in vitro and in vivo studies involving various types of cancer. Indeed, 2-AEH2P has modulated proliferative and apoptotic effects in human breast cancer cells with estrogen, progesterone, and glucocorticoid receptors (MCF-7) [24], in triple-negative breast cancer cells (MDA-MB-231) [25], murine melanoma (B16-F10) [26], K-562 and K-562 MDR(+) myeloid leukemia [27], and murine hepatoma (Hepa-1c1c7) [28], indicating significant data in these cell lines regarding the reduction of mitochondrial membrane potential and the expression of markers involved in cell death, angiogenesis, and proliferation. In turn, paclitaxel (PTX) is a widely used chemotherapy agent in the treatment of advanced metastatic breast cancer [29]. Unlike antimitotic chemotherapeutics such as cisplatin (platinum) and doxorubicin (anthracycline), which act by interacting with DNA, interfering with its replication [30], and stabilizing microtubules, preventing their disassembly in the metaphase, resulting in mitotic arrest and apoptosis [31,32,33,34,35,36,37,38,39,40], there exists resistance in tumors, which is primarily associated with the overexpression of transporters that block drug accumulation within tumor cells and prevent their binding to their target [35,36]. In this context, it is interesting to investigate whether the combinatorial action of 2-AEH2P with Simvastatin, GM-CSF, Coenzyme Q10, and the chemotherapeutic paclitaxel can have a pharmacological effect, enhancing pro-apoptotic activity. To assess this, the Ehrlich ascitic carcinomatosis (EAT) model was used. Peritoneal carcinomatosis in breast cancer develops most frequently when tumor cells spread throughout the peritoneal cavity, leading to multiple tumors, the clinical significance of which is a tumor with a poor prognosis [41]. Furthermore, its frequency occurs more in women and is related to several factors, such as age, family history of ovarian or peritoneal cancer, BCRA genetic mutations, hormone replacement therapy, obesity, and endometriosis [41]. In turn, the ascitic model of Ehrlich’s tumor acts on the membrane of the animal’s peritoneal cavity. The EAT model represents a multifaceted preclinical tumor model that is applied in pathophysiological and pharmacological screening studies for cancers [42]. This is not limited to classic neoplasms, but also extends to systemic involvement, generating conditions such as hepatotoxicity and higher levels of neoplastic markers, such as α-fetoprotein and carcinoembryonic tumor antigen, frequently observed in oncology practice; in this experimental model, the tumor develops according to its vascularization and the increase in vascularization is associated with its rapid growth rate [43,44,45]. Increased vascularity is associated with its rapid growth rate. Its wide use in experimental oncology to investigate the therapeutic capacity of different synthetic chemotherapy drugs or to evaluate the antitumor activity of different substances of natural origin is related to its ability to mimic processes of metastasis, angiogenesis, and immune response associated with tumors [37]. Furthermore, its aggressiveness and resistance to cell death are associated with the absence of the estrogen receptor (ER-) [38] and the overexpression of HSP70 [39,40], a chaperone system protein associated with lymph node metastases, decreased survival, and resistance to chemotherapy in breast cancer [46].

## 2. Methods

### 2.1. Acquisition of Ehrlich Ascitic Carcinoma (EAT) Cells

Ehrlich Ascitic Carcinoma, described by Paul Ehrlich in 1905, is a spontaneous mammary adenocarcinoma that develops in mice. Tumor cells are maintained by intraperitoneal (ip) passages in ascitic form, which is used for pharmacological screening and pathophysiological investigation [47,48]. Female Balb/c mice were anesthetized with Xylazine (10–15 mg/kg) administered intraperitoneally as a single dose, and Ketamine (100–150 mg/kg) administered intraperitoneally as a single dose. Tumor induction was performed by injecting tumor cells intraperitoneally at a concentration of 2 × 10^4^ for development. In vivo maintenance of tumor cells involved successive passages of tumor cells from mice with ascitic tumors on the 14th day to healthy mice. Mouse handling followed the standards established by the Standard Operating Procedures of the Ethics Committee for Animal Use at Instituto Butantan (CEUAI), the ethical principles of animal experimentation of the Brazilian College of Animal Experimentation (COBEA), and the Canadian Council on Animal Care (CCAC).

### 2.2. Cell Culture

In vitro Ehrlich tumor cells were maintained in RPMI culture medium (Cultilab, Campinas, Brazil) containing 10% fetal bovine serum (FBS), 200 mM sodium bicarbonate, pH = 7.4, in a humidified incubator with 5% CO_2_ at 37 °C, and cell counts were performed using a Neubauer chamber before experiments. The trypan blue test was conducted to ensure optimal cell viability (greater than 94%) for experiments. Normal murine fibroblast cells (L929), obtained from the American Type Cell Collection (ATCC CCL-1^TM^ code), were thawed and transferred to a cell culture flask (25 cm^2^), containing RPMI 1640 culture medium (Cultilab, Campinas, Brazil) supplemented with 10% FBS, 200 mM sodium bicarbonate, pH = 7.4, in an incubator with 5% CO_2_ at 37 °C. When arranged in monolayers for experiments, they underwent enzymatic dissociation with 0.2% trypsin + 0.02% EDTA solution for detachment and were neutralized by adding RPMI culture medium containing 10% FBS.

### 2.3. Determination of Cytotoxic Activity Using the MTT Method

Normal Ehrlich ascitic tumor (EAT) cells and murine fibroblasts (L929) were incubated in 96-well plates at a concentration of 1 × 10^5^ cells per well for 24 h.

After the addition of the compounds individually or in combination for 24 h, the supernatant was collected, and 100 µL of MTT (Calbiochem—Darmstadt, Germany) at a concentration of 5 mg/mL was added and incubated for 3 h in a CO_2_ incubator at 37 °C. At the end of this period, the contents were removed, and 100 µL of methyl alcohol was added to dissolve the precipitated formazan crystals. Absorbance quantification was performed using an ELISA reader at a wavelength of 540 nm. The concentration that induces toxicity in 50% of the cells (IC50%) was determined after 24 h of treatment at different concentrations. All tests were performed with three independent repetitions to assess the dose-response effect. The control was established with Ehrlich ascitic tumor cells and normal murine fibroblast cells (L929) containing only RPMI-1640 medium. Treatments that were conducted in association with 2-AEH2P at a fixed concentration half of the IC50 value, previously determined by the same method, were added 3 h before the administration of 2-AEH2P. In addition, they were evaluated at different concentrations by serial dilution to sensitize tumor and normal cells, containing only 2-AEH2P at a fixed concentration of 22.8 mM. IC50 values were obtained using GraphPad Prism 5 (GraphPad Software Inc., San Diego, CA, USA). After establishing the values for the “X” and “Y” axes, the data were normalized to a percentage (100%). Concentration values were converted into logarithmic values, followed by linear regression. To open the “Dose-Response—Inhibition” equations, choose “log (inhibitor) vs. response”. In the bottom part of the dialog box, select the option “Interpolate unknowns from the standard curve”, and the IC50 values are obtained.

### 2.4. Analysis of Cell Cycle Phases and Fragmented DNA by Flow Cytometry

Normal and tumor cells were treated for a period of 24 h. For their evaluation associated with 2-AEH2P, the same time conditions between drug administrations described in Section 2.3 were applied. In turn, the concentrations (Table 1) evaluated were also defined according to the values obtained for EAT tumor cells indicated by the test described in Section 2.3.

The cells were trypsinized and centrifuged at 1500 rpm for 5 min. Subsequently, the pellet was suspended in a solution of 70% ethanol and RNase and stored in a −20 °C freezer for 24 h. The samples were centrifuged at 1500 rpm for 5 min and resuspended in 200 µL of FACS buffer, 20 µL of Triton X-100 (Sigma-Aldrich, Burlington, MA, USA), and 50 µg/mL propidium iodide (Sigma-Aldrich). They were kept for 30 min at room temperature, protected from light. After this period, the samples were transferred to cytometry tubes and taken for analysis on a FACSCalibur flow cytometer (BD) at FL-1 fluorescence intensity. The acquired histograms were analyzed using the Cell-Quest program (BD).

### 2.5. Analysis of Mitochondrial Membrane Potential

Mito-Red (Sigma-Aldrich) is a specific fluorochrome for labeling active mitochondria. Tumor and normal cell samples, when associated with 2-AEH2P, were treated with drug administration intervals of 3 and 12 h, considering the concentrations described in Table 1. Both cell lines, after 24 h treatment, were centrifuged at 1500 rpm for 5 min, the supernatant was discarded, and 100 µL of RPMI 0.1% + Mito-Red medium (Sigma-Aldrich) was added. The samples were then kept in incubators with 5% CO_2_ at 37 °C for 1 h. After this period, the tubes were centrifuged, the supernatant discarded, and the pellet was suspended in 100 µL of FACS Flow buffer. The reading and analysis of Mito-Red labeling were performed on a Facscanto^®^ flow cytometer (BD, Franklin Lakes, NJ, USA) at FLH-1 fluorescence intensity. Histograms were acquired and analyzed using the Cell-Quest-BD program.

### 2.6. Evaluation of Cell Marker Expression by Flow Cytometry

Tumor cells, when associated with 2-AEH2P, were sensitized for a period of 3 and 12 h, as described in Section 2.5. After treatments, at the concentrations presented in Table 1, for intracytoplasmic markers, cells were permeabilized with 0.1% Triton-X solution for 30 min at room temperature. Markers involved in the promotion and control of proliferation, inflammation, and cell death (Bcl-2, Caspase 3, Cytochrome C, p53, TNF-α) were utilized. After this period, cells were centrifuged at 1500 rpm and washed with chilled PBS. The supernatant was discarded, and the sediment was suspended in 200 µL of FACS Flow buffer containing 0.1% paraformaldehyde. The FACScanto^®^ flow cytometer (BD) was used for acquisition and analysis of receptor expression on the cell surface of tumor cells at FL-1 fluorescence intensity. Histograms were acquired and analyzed using the Cell-Quest-BD program.

### 2.7. Analysis of Pharmacological Effect and Drug Combination

To determine the potential synergistic effect of associated treatments in both cell lines, the Synergy Finder 2.0 software quantified the degree of synergy over the multiplicative effects of single drugs independently, based on the Bliss independence model. The overall combination mean or synergy score (x) indicates that: X < −10 has an antagonistic effect, X ≤ 10 or X ≥ −10 has an additive effect, and X > 10 has a synergistic effect.

$$\begin{array}{l} S_{\mathrm{BLISS}}=E_{A,B}-\left(E_{A},+,E_{B}\right)\\ \mathrm{Synergy}\;\mathrm{score}=\frac{-log\;\left(p\right)}{log\;\left(0.05\right)}\times \frac{t}{\left|t\right|} \end{array}$$

### 2.8. Statistical Analysis

The obtained values were expressed as mean ± standard deviation from three independent experiments, and after obtaining the individual values for each cell line, the results were tabulated and analyzed using GraphPad software, version 7.0, and Instant Pad Prism version 5.0. Data analysis was performed by comparing two or more groups with non-parametric distribution using analysis of variance (ANOVA), followed by the Tukey–Kramer multiple comparisons test, with the critical level considered for significance being *p* ≤ 0.05.

## 3. Results

### 3.1. Analysis of the Compound 2-AEH2P in Murine Fibroblast Cells (L929) and Ehrlich Ascitic Tumor (EAT) Cells

**Figure 1:**
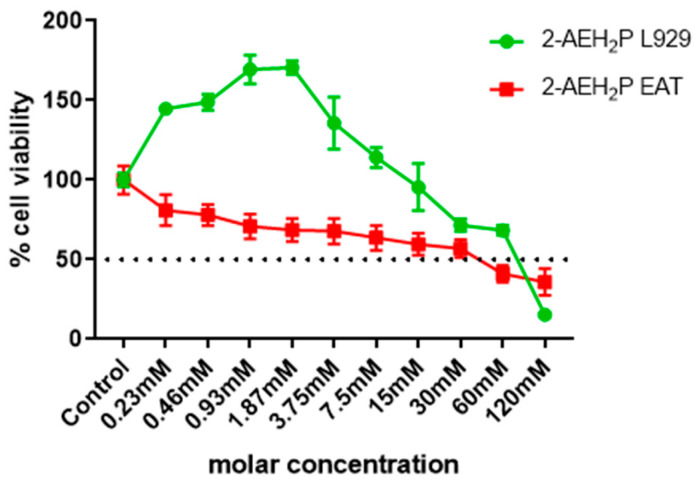
Cytotoxicity determination in normal murine fibroblastic cells (L929)—IC50% 54.9 mM, and Ehrlich Ascitic Tumor (EAT) cells—IC50% 45.6 mM, by the MTT method treated with 2-AEH2P. Values are expressed as mean ± SD of three independent experiments. Statistical differences were obtained by the non-parametric ANOVA variance test and Tukey–Kramer multiple comparisons test.

**Figure 2:**
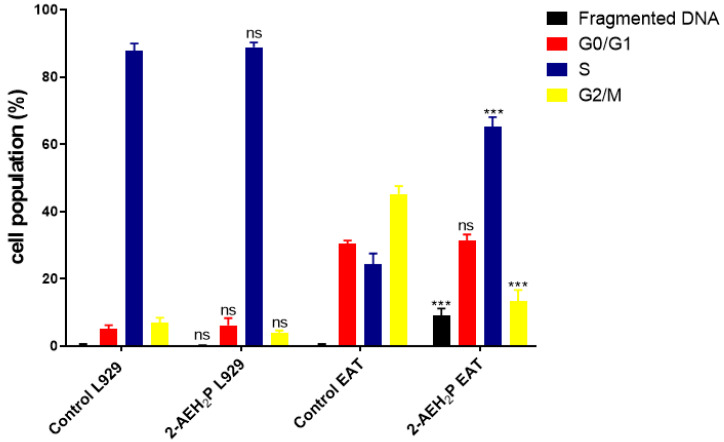
Analysis of cell cycle phases in normal murine L929 fibroblast cells and Ehrlich Ascitic Tumor (EAT) cells treated with 2-AEH2P at a concentration of 45.6 mM. Representative histograms of cell distribution in cell cycle phases and DNA fragments, bar graph showing the correlation of the cell cycle effect expressed as mean ± SD of three independent experiments. Significance levels: ns = not significant, *** *p* < 0.001.

**Figure 3:**
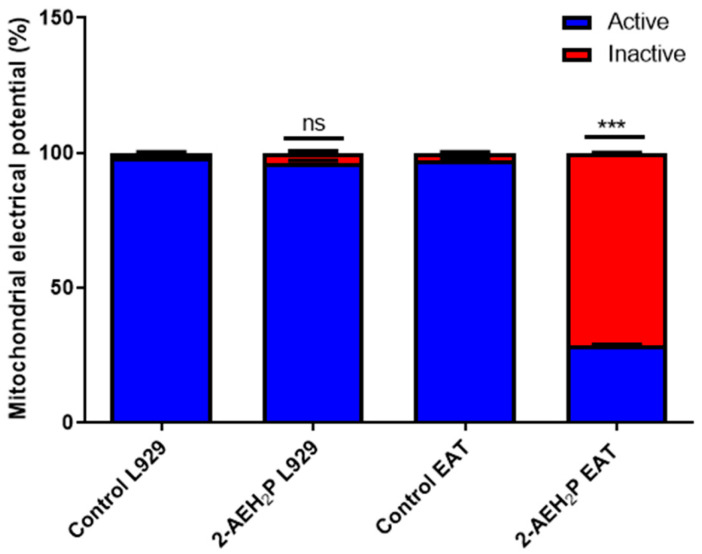
Mitochondrial membrane potential graph. Normal murine L929 fibroblastic cells and Ehrlich Ascitic Tumor (EAT) cells treated with 2-AEH2P at a concentration of 45.6 mM. Bar graph showing the correlation of the treatment effect on mitochondria expressed as mean ± SD of three independent experiments. Significance levels: ns = not significant, *** *p* < 0.001.

**Figure 4:**
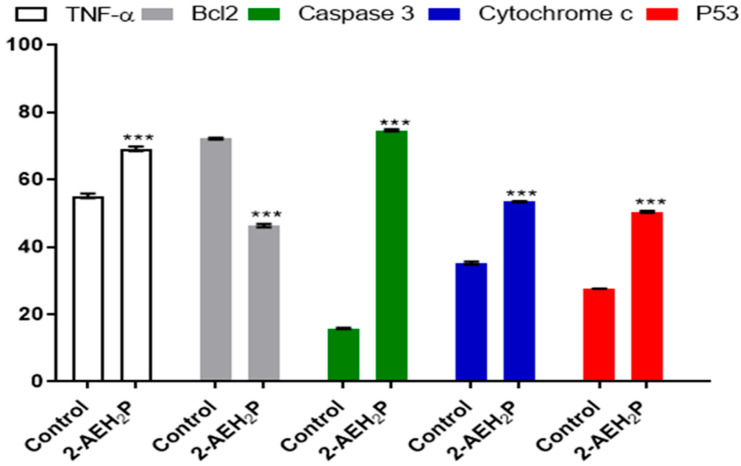
Graph of the expression of TNF-α/DR-4, Bcl2, Caspase 3, cytochrome c, and P53 markers in Ehrlich ascitic tumor cells treated with 2-AEH2P at a concentration of 45.6 mM, quantified by flow cytometry, 24 h after treatment. Values are expressed as mean ± SD of three independent experiments. Statistical differences were obtained by the non-parametric ANOVA variance test and Tukey–Kramer multiple comparisons test. Significance levels: *** *p* < 0.001.

The determination of cytotoxicity based on the 50% inhibitory concentration (IC50%) indicated that tumor cells reduced their activity by half at a concentration of 45.6 mM. In contrast, normal cells showed an IC50% of 54.9 mM (Figure 1). Interestingly, it was also observed that at lower concentrations of 2-AEH2P, normal murine fibroblast cells (L929) exhibited a proliferative effect (Figure 1). It is essential to note that the following results were obtained by evaluating the response of both cell lines (L929 and EAT) subjected to the IC50% concentration of 45.6 mM, indicated for Ehrlich ascitic tumor (EAT) cells. Regarding the ability to model the distribution profile of cell populations in cell cycle phases, normal L929 fibroblast cells, compared to the control group, showed no significant difference (Figure 2). However, in EAT tumor cells compared to the control group, when treated with 2-AEH2P, respective changes in cell cycle phases were observed (Figure 2). There was an increase of 9.01 ± 2.2% in fragmented DNA, a reduction of 13.5 ± 3.2% in the cell population in the G2/M phase, and an increase in cells in the S phase with a percentage of 65.2 ± 2.9%. It is worth noting that, for EAT tumor cells in the control group, the distribution of cell populations indicated respective percentages of 45.3 ± 2.3% and 24.3 ± 3.3% for the G2/M and S phases. Regarding the mitochondrial membrane potential (Figure 3), it was observed that there were no significant differences in the mitochondrial membrane potential for normal L929 fibroblast cells evaluated at this concentration. However, EAT tumor cells exhibited a significant decrease in their mitochondrial membrane potential to 29.9 ± 1.1%. Furthermore, the expression of pro-apoptotic markers, TNF-α, Caspase-3, and P53, was increased in Ehrlich ascitic tumor cells compared to their control group (Figure 4). Similarly, and according to the results, it is observed that the pro-apoptotic action of 2-AEH2P is likely related to the destabilization of the mitochondria, considering the decrease in the expression of the anti-apoptotic protein Bcl2 and the increase in the expression of Cytochrome c (Figure 4).

### 3.2. Analysis of the Compound 2-AEH2P Associated with Simvastatin in Murine Fibroblast Cells (L929) and Ehrlich Ascitic Tumor (EAT) Cells

**Figure 5:**
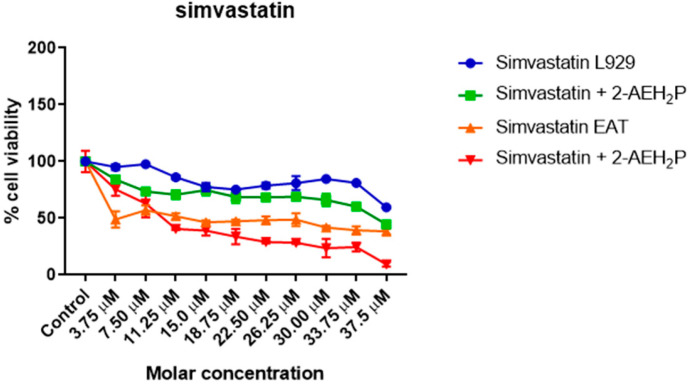
Determination of cytotoxicity in normal murine fibroblastic cells (L929) and Ehrlich ascitic tumor (EAT) cells using the colorimetric MTT method treated with Simvastatin (serial dilution) and Simvastatin (serial dilution) + 2-AEH2P (22.8 mM). Values expressed as mean ± SD from three independent experiments. Statistical differences were obtained using the non-parametric ANOVA variance test and Tukey–Kramer multiple comparisons test.

**Figure 6:**
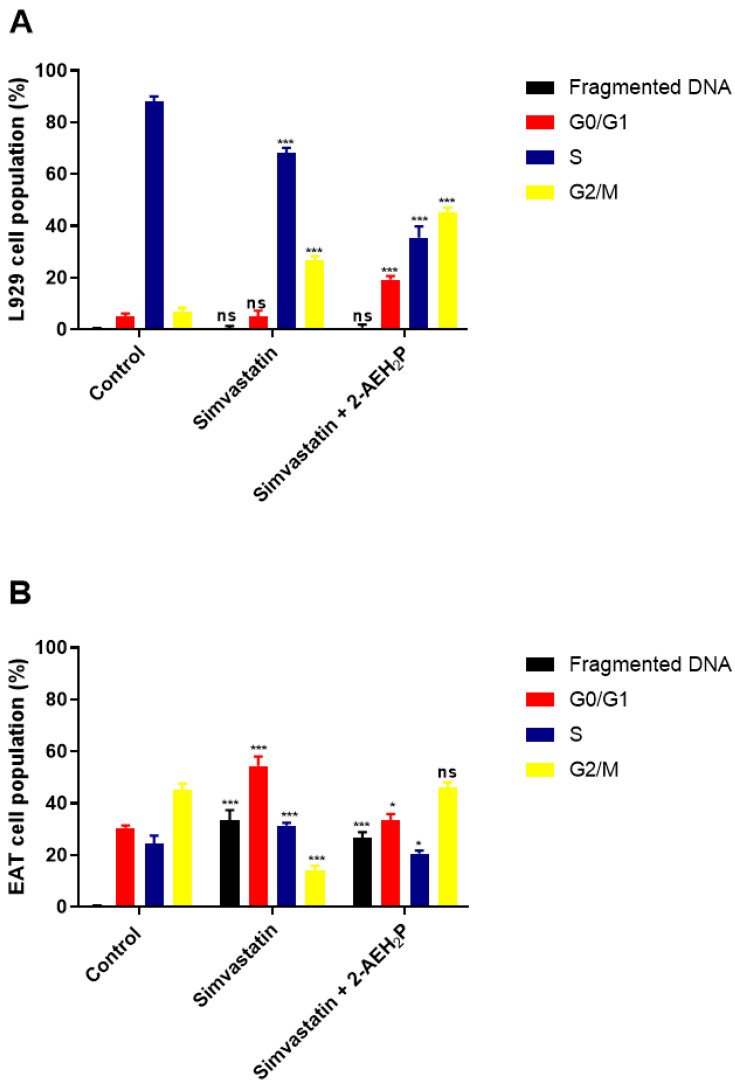
Analysis of cell cycle phases. (**A**) Normal murine fibroblastic cells (L929); (**B**) Ehrlich ascitic tumor (EAT) cells. Cells were treated with Simvastatin (9.6 µM) and Simvastatin (10.1 µM) + 2-AEH2P (22.8 mM), for a period of 24 h. Representative histograms of cell distribution in cell cycle phases and fragmented DNA, and bar graph showing the correlation of the cell cycle effect expressed as mean ± SD from three independent experiments. Significance levels: ns = not significant, * *p* < 0.01, and *** *p* < 0.001.

**Figure 7:**
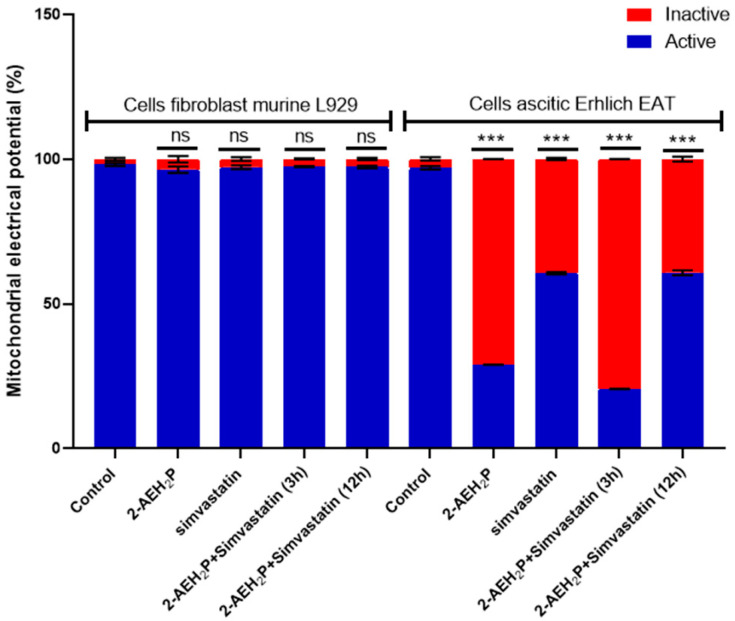
Mitochondrial membrane potential. Normal murine fibroblast cells (L929) and Ehrlich ascitic tumor (EAT) cells were treated with 2-AEH2P (45.6 mM), Simvastatin (9.6 µM), and Simvastatin (10.1 µM) + 2-AEH2P (22.8 mM), for a period of 24 h. Bar graph showing the correlation of the treatment effect on mitochondria expressed as mean ± SD from three independent experiments. Significance levels: ns = not significant, *** *p* < 0.001.

**Figure 8:**
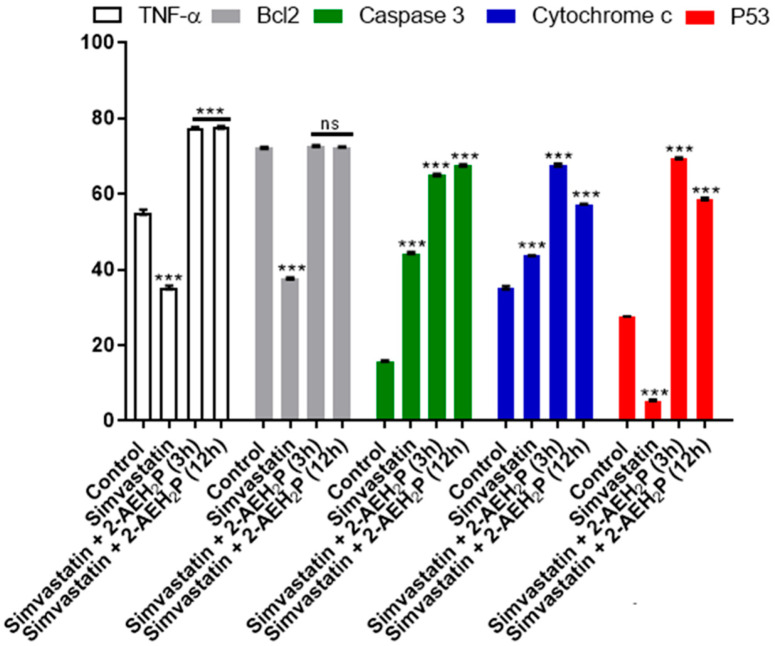
Graph of the expression of TNF-α/DR-4, Bcl2, Caspase 3, cytochrome c, and P53 markers in Ehrlich ascitic tumor cells treated with Simvastatin (9.6 µM) and Simvastatin (10.1 µM) + 2-AEH2P (22.8 mM). Quantification by flow cytometry, 24 h after treatments. Values expressed as mean ± SD from three independent experiments. Statistical differences were obtained by non-parametric ANOVA and Tukey–Kramer multiple comparisons test. Significance levels: ns = not significant, *** *p* < 0.001.

**Figure 9:**
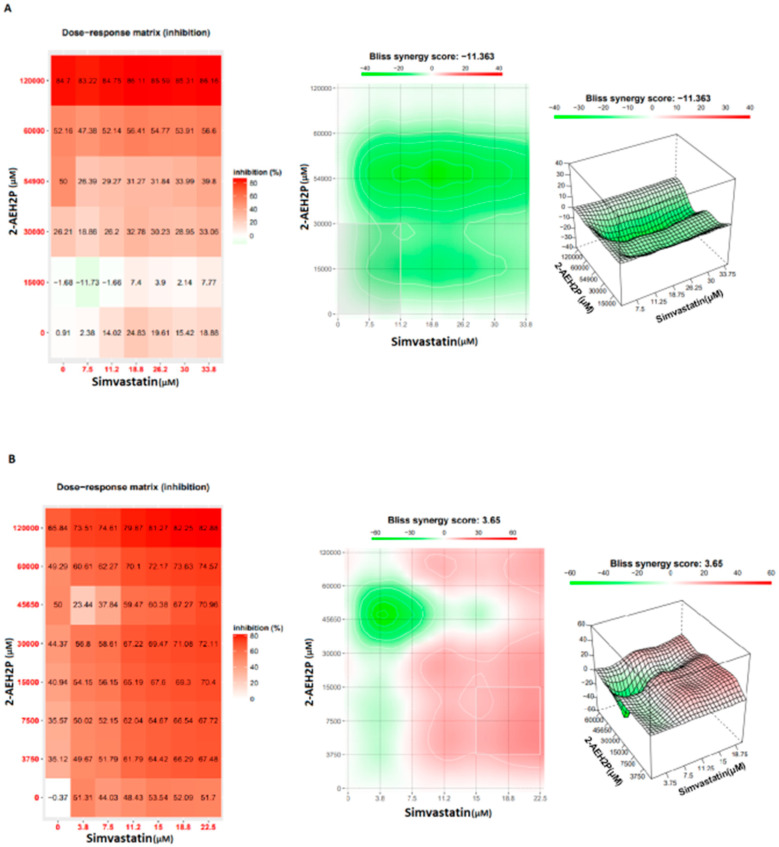
Analysis of the degree of pharmacological interactions. (**A**) Normal murine fibroblast cells (L929); (**B**) Ehrlich ascitic tumor (EAT) cells. Treated with varying concentrations of Simvastatin, 2-AEH2P, and Simvastatin + 2-AEH2P combination for 24 h. Cell viability results were subjected to analysis using the Bliss independence model, SynergyFinder. The Bliss independence model logarithmically calculated the expected combinatorial effect based on the probability of independent events caused by the drug combination.

The evaluation of inhibitory concentrations (IC50%) for murine L929 fibroblasts and Ehrlich ascitic tumor cells treated with Simvastatin showed IC50% concentrations of 109 μM and 9.6 μM, respectively (Figure 5). When subjected to the same analysis again, associated with 2-AEH2P, both cell lines were sensitized in a serial dilution for Simvastatin and subsequently supplemented with 2-AEH2P at half its IC50% concentration, 22.8 mM. This value comes from the previously established treatment for EAT tumor cells. It is worth noting that all other combinations followed this pattern of serial dilution and a fixed concentration of 22.8 mM for 2-AEH2P. Thus, it was observed that the association of Simvastatin + 2-AEH2P for normal L929 cells reduced its IC50% to 63 μM. However, for tumor cells, the IC50% of 10.1 μM remained close to its isolated value (Figure 5). For cell cycle assays and quantification of mitochondrial potential, both cell lines were treated with Simvastatin 9.6 μM and Simvastatin (10.1 μM) + 2-AEH2P (22.8 mM). There is modulation of the cell cycle in both cell lines subjected to treatments. In normal L929 cells, both treatments significantly reduced the cell population in the S phase, while an increase in the cell population in the G2/M phase was observed (Figure 6A). However, no fragmented DNA was observed. Similarly, there was significant modulation of the cell cycle phases in tumor cells, with both treatments showing an increase in the G0/G1 phase and exhibiting the presence of fragmented DNA. Tumor cells treated only with Simvastatin also showed an increase in the S phase compared to the control group and a decrease in the G2/M phase (Figure 6B). Cells treated with the combination indicated a lower degree of significance in the modulation of the G0/G1 and S phases in the cell cycle compared to the control and treatment with Simvastatin alone (Figure 6B). Regarding mitochondrial membrane potential, unlike normal L929 cells that showed no modification in their membrane potential, all treatments indicated a significant reduction in their potential. However, EAT tumor cells sensitized for 3 h with Simvastatin associated with 2-AEH2P showed better results compared to other treatments (Figure 7). The analysis of the expression of cell markers for tumor cells, for treatment only with Simvastatin, indicated a significant increase in the expression of Caspase 3 and Cytochrome c, and a decrease in Bcl2. However, there was also a significant reduction in TNF-α and P53 (Figure 8). When treated in association with 2-AEH2P, in both situations of previous sensitivity (3 h and 12 h), tumor cells did not show a reduction in the expression of Bcl2. However, a significant increase in the expression of TNF-α, Caspase 3, Cytochrome c, and P53 was identified. Furthermore, the synergy profile analysis between these associations indicated a synergistic score of −11.36 for L929 (Figure 9A), compared to EAT tumor cells that showed a synergistic score of 3.65 (Figure 9B). It is observed that under the conditions of treatment with Simvastatin (10.1 μM) + 2-AEH2P (22.8 mM), the association exhibits a greater additive effect for tumor cells.

### 3.3. Analysis of the Compound 2-AEH2P associated with GM-CSF in Normal Murine Fibroblast Cells (L929) and Ehrlich Ascitic Tumor (EAT) Cells

**Figure 10:**
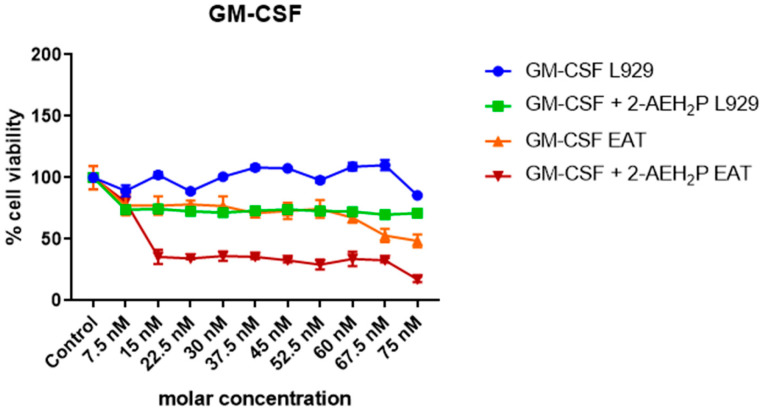
Determination of cytotoxicity in normal murine fibroblastic cells (L929) and ascitic Ehrlich tumor (EAT) cells by the colorimetric MTT method treated with GM-CSF (serial dilution) and GM-CSF (serial dilution) + 2-AEH2P (22.8 mM). Values are expressed as mean ± SD from three independent experiments. Statistical differences were obtained by the non-parametric ANOVA variance test and Tukey–Kramer multiple comparisons test.

**Figure 11:**
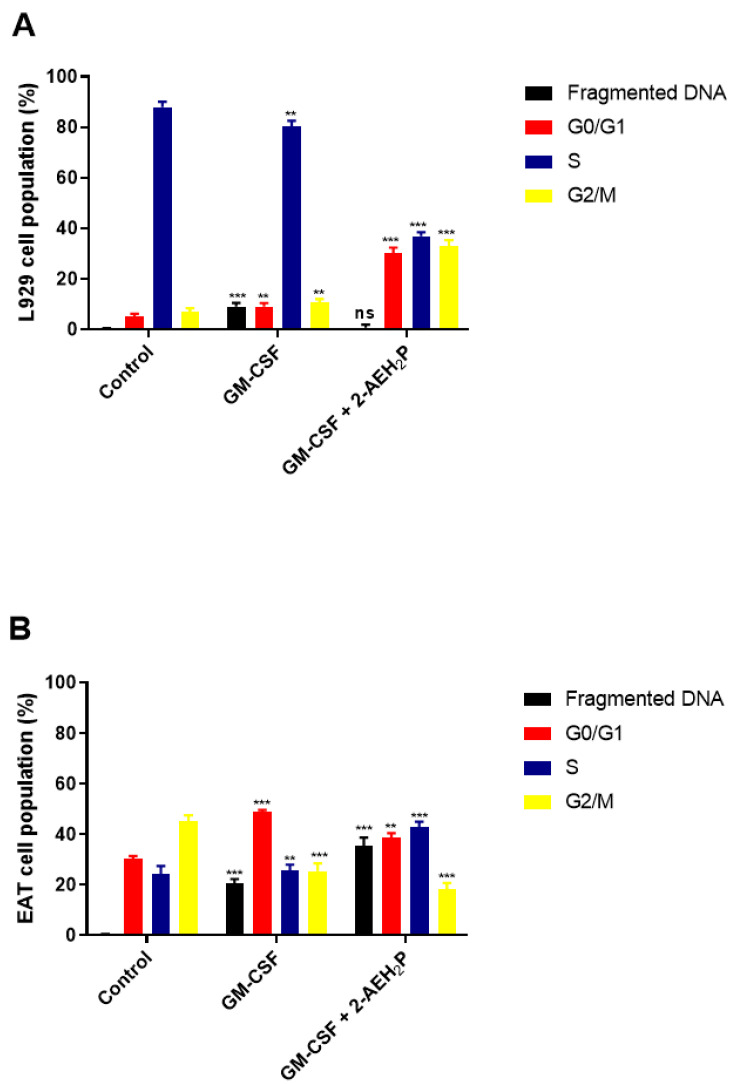
Analysis of cell cycle phases. (**A**) Normal murine fibroblasts (L929); (**B**) Ehrlich ascitic tumor (EAT) cells. Cells were treated with GM-CSF (144.1 nM) and GM-CSF (16 nM) + 2-AEH2P (22.8 mM) for a period of 24 h. Representative histograms of cellular distribution in cell cycle phases and fragmented DNA, and bar graph showing correlation of the cell cycle effect expressed as mean ± SD from three independent experiments. Significance levels: ns = not significant, ** *p* < 0.01, and *** *p* < 0.001.

**Figure 12:**
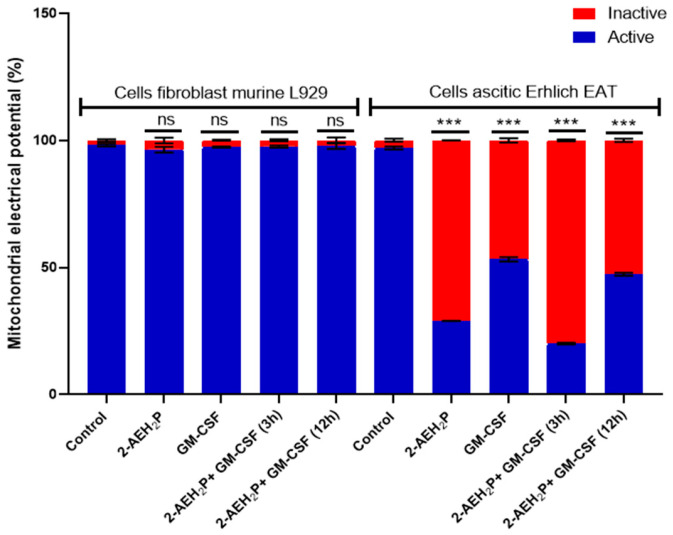
Mitochondrial electrical potential. Normal murine fibroblasts (L929) and Ehrlich ascitic tumor (EAT) cells were treated with 2-AEH2P (45.6 Mm), GM-CSF (144.1 nM), and GM-CSF (16 nM) + 2-AEH2P (22.8 mM) for a period of 24 h. Bar graph showing the correlation of the treatment effect on mitochondria expressed as mean ± SD from three independent experiments. Significance levels: ns = not significant, *** *p* < 0.001.

**Figure 13:**
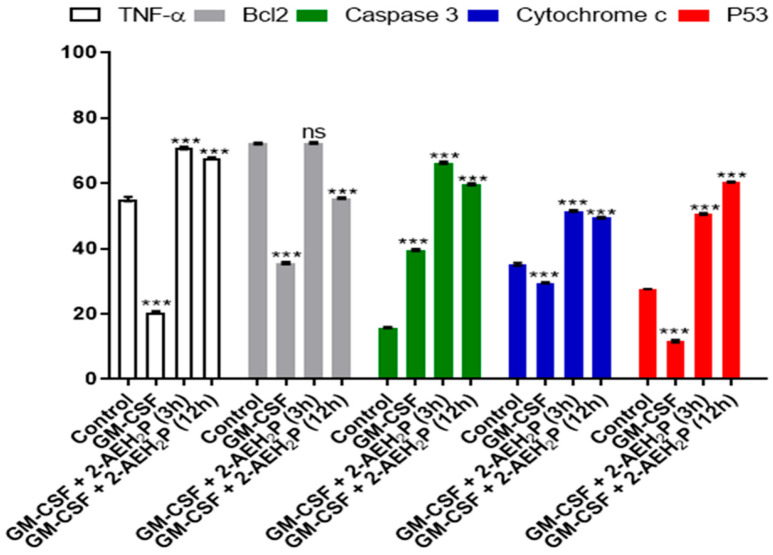
Graph of the expression of markers TNF-α/DR-4, Bcl2, Caspase 3, cytochrome c, and P53 in Ehrlich ascitic tumor cells treated with GM-CSF (144.1 nM) and GM-CSF (16 nM) + 2-AEH2P (22.8 mM). Quantification by flow cytometry, 24 h after treatments. Values expressed as mean ± SD from three independent experiments. Statistical differences were obtained by the non-parametric variance ANOVA test and Tukey–Kramer multiple comparisons test. Level of statistical significance: ns = not significant, *** *p* < 0.001.

**Figure 14:**
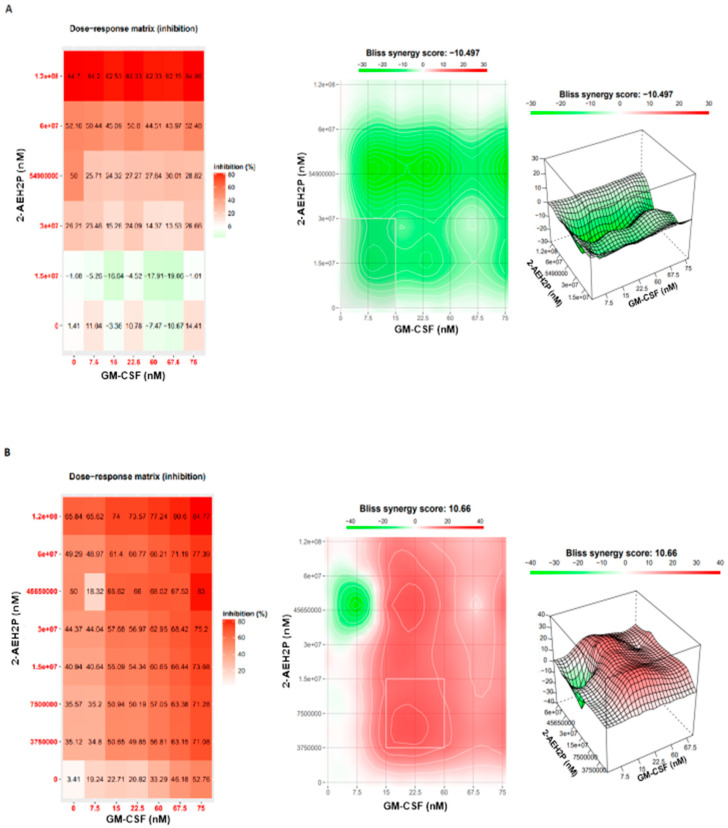
Analysis of the degree of pharmacological interactions. (**A**) Normal murine fibroblast cells (L929); (**B**) Ehrlich ascitic tumor (EAT) cells. Treated, at various concentrations, with GM-CSF, 2-AEH2P, and GM-CSF + 2-AEH2P for 24 h. Cell viability results were analyzed using the Bliss method and SynergyFinder. The Bliss independence model logarithmically calculated the expected combinatorial effect based on the probability of independent events caused by the combination of drugs.

No toxicity was observed for normal murine fibroblasts L929 treated with GM-CSF or GM-CSF + 2-AEH2P (22.8 mM) when subjected to IC50% analysis (Figure 10). However, for EAT tumor cells, an IC50% of 144.1 nM was identified in the treatment with GM-CSF, and its association with 2-AEH2P (22.8 mM) quantitatively reduced its IC50% to 16 nM (Figure 10). In turn, in both cell lines subjected to treatments with GM-CSF (144.1 nM) and GM-CSF (16 nM) + 2-AEH2P (22.8 mM), modulations in the cell cycle were observed (Figure 11). However, EAT tumor cells exhibited a higher percentage of DNA fragments (Figure 11B) compared to their control and normal murine fibroblasts L929 treated only with GM-CSF (Figure 11A). In addition, it was possible to observe that EAT tumor cells decreased their percentage of cells in the G2/M phase compared to the control group. As described in the analysis of the sinvastatin treatment, no modifications were observed in the mitochondrial potential of normal cells subjected to treatment with GM-CSF (144.1 nM) and GM-CSF (16 nM) + 2-AEH2P (22.8 mM). However, for tumor cells, a significant decrease occurred in all treatments. The GM-CSF treatment for 3 h + 2-AEH2P showed the greatest decrease in electrical potential when associated and compared to the control or the group treated only with 2-AEH2P or GM-CSF (Figure 12). In contrast, the group treated with GM-CSF (3h) + 2-AEH2P was the only one that did not show a significant decrease in the expression of Bcl2, although it increased the expression of TNF-α/DR-4, Caspase 3, Cytochrome c, and P53 (Figure 13). The analysis of pharmacological combinations indicated an antagonistic effect with a score of −10.50 for normal murine fibroblast cells L929 (Figure 14A). In contrast, the same combination when treating tumor cells indicated a synergistic effect with a score of 10.66 (Figure 14B).

### 3.4. Analysis of the Compound 2-AEH2P Associated with Coenzyme Q10 in Normal Murine Fibroblast Cells (L929) and Ehrlich Ascitic Tumor (EAT) Cells

**Figure 15:**
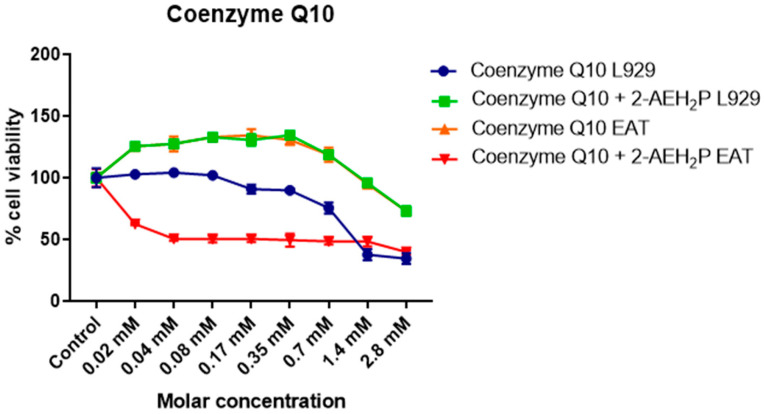
Determination of cytotoxicity in normal murine fibroblastic cells (L929) and Ehrlich ascitic tumor (EAT) cells using the colorimetric MTT method treated with Coenzyme Q10 (serial dilution) and Coenzyme Q10 (serial dilution) + 2-AEH2P (22.8 mM). Values expressed as mean ± SD from three independent experiments. Statistical differences were obtained by non-parametric ANOVA variance test and Tukey–Kramer multiple comparisons test.

**Figure 16:**
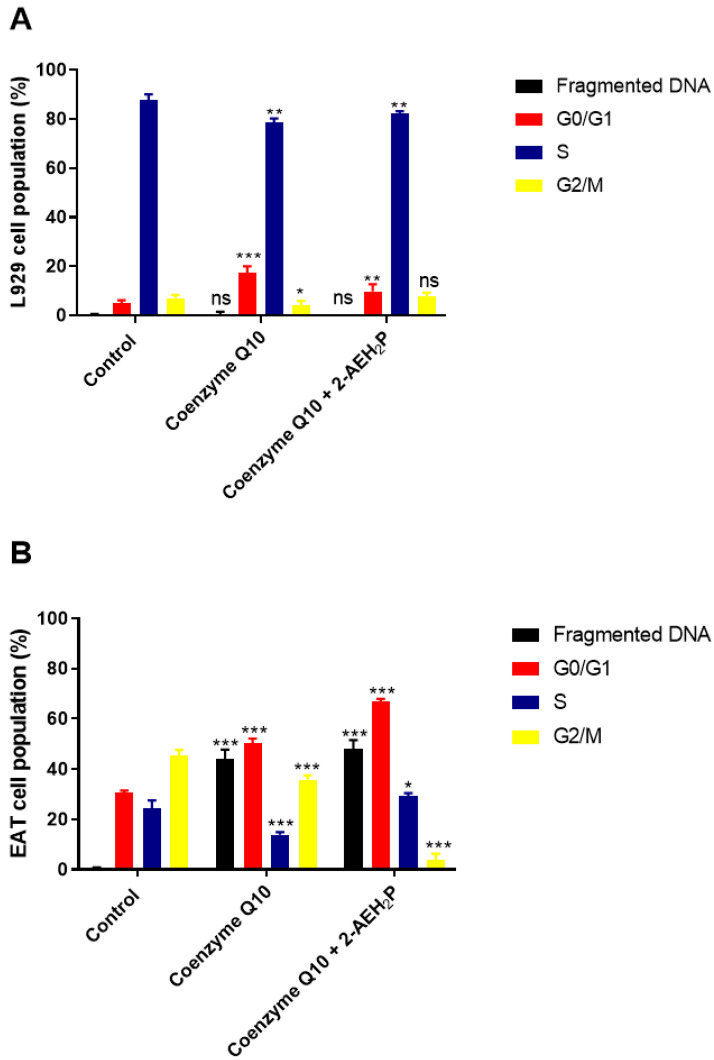
Analysis of cell cycle phases. (**A**) Normal murine fibroblasts (L929); (**B**) Ehrlich ascitic tumor (EAT) cells. Cells were treated with Coenzyme Q10 (5.5 μM) and Coenzyme Q10 (5.8 μM) + 2-AEH2P (22.8 mM) for 24 h. Representative histograms of cellular distribution in cell cycle phases and fragmented DNA, and bar graph showing correlation of cell cycle effect expressed as mean ± SD from three independent experiments. Significance levels: ns = not significant, * *p* < 0.05, ** *p* < 0.01, *** *p* < 0.001.

**Figure 17:**
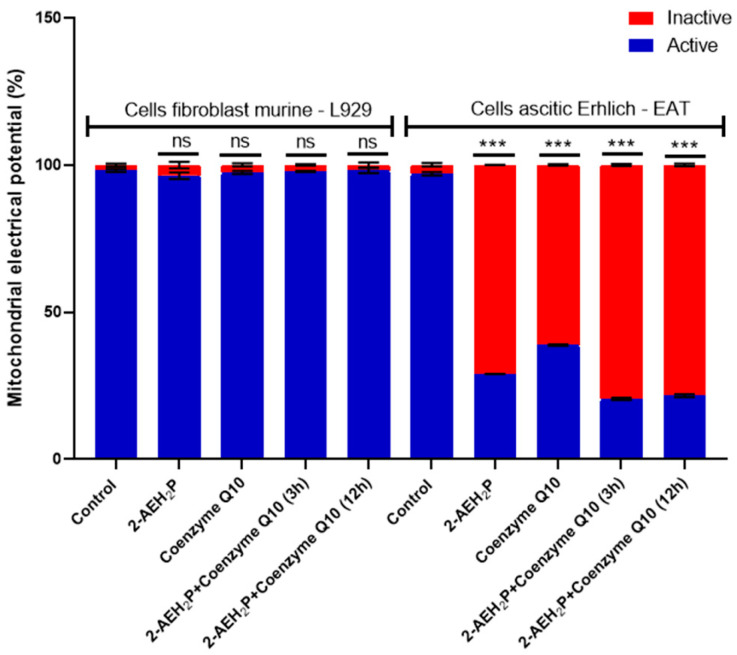
Mitochondrial electric potential. Normal murine fibroblasts (L929) and Ehrlich ascitic tumor (EAT) cells were treated with 2-AEH2P (45.6 Mm), Coenzyme Q10 (5.5 μM), and Coenzyme Q10 (5.8 μM) + 2-AEH2P (22.8 mM) for a period of 24 h. Bar graph showing the correlation of the treatment effect on mitochondrial electric potential expressed as mean ± SD from three independent experiments. Significance levels: ns = not significant, *** *p* < 0.001.

**Figure 18:**
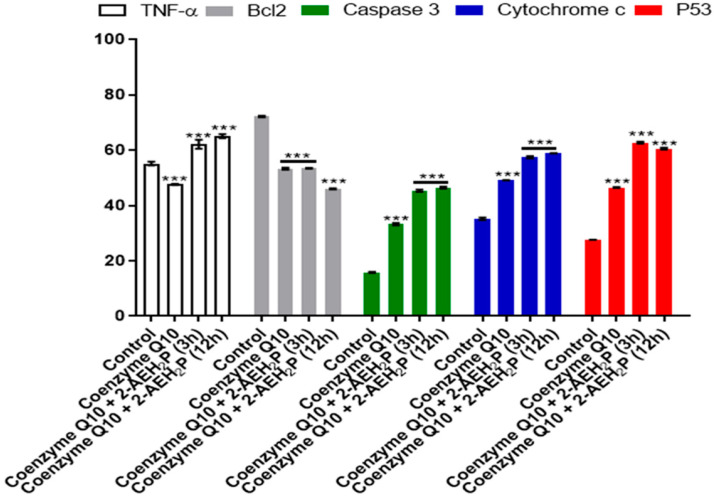
Graph of the expression of markers TNF-α/DR-4, Bcl2, Caspase 3, cytochrome c, and P53 in Ehrlich ascitic tumor cells treated with Coenzyme Q10 (5.5 μM) and Coenzyme Q10 (5.8 μM) + 2-AEH2P (22.8 mM). Quantification by flow cytometry, 24 h after treatments. Values expressed as mean ± SD from three independent experiments. Statistical differences were obtained by the non-parametric ANOVA variance test and Tukey–Kramer multiple comparisons test. Significance levels: *** *p* < 0.001.

**Figure 19:**
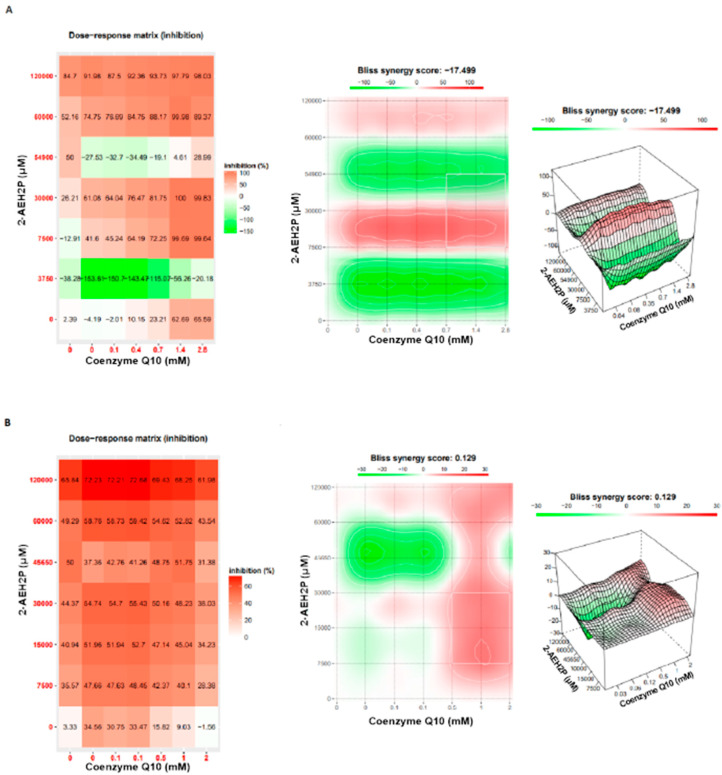
Analysis of the degree of pharmacological interactions. (**A**) Normal murine fibroblast cells (L929); (**B**) Ehrlich ascitic tumor (EAT) cells. Treated with varying concentrations of Coenzyme Q10, 2-AEH2P, and Coenzyme Q10 + 2-AEH2P for 24 h. The results of cell solutions were subjected to analysis using the Bliss independence model and SynergyFinder. The Bliss independence model logarithmically calculated the expected combinatorial effect based on the probability of independent events caused by the combination of drugs.

Coenzyme Q10 exhibited an IC50% of 2.8 mM for normal L929 fibroblasts (Figure 15). However, its association with 2-AEH2P did not show a toxic effect. Nevertheless, at lower concentrations, it was observed that individually and in combination with 2-AEH2P, EAT tumor cells, respectively, exhibited IC50% values of 5.5 μM and 5.8 μM (Figure 15). When evaluating the distribution of cell cycle phases (Figure 16A,B), no DNA fragments were observed in normal L929 fibroblasts, although significant alterations in their distribution were noted for both Coenzyme Q10 (5.5 μM) and Coenzyme Q10 (5.8 μM) + 2-AEH2P (22.8 mM) treatments (Figure 16A). On the other hand, EAT tumor cells for both treatments exhibited DNA fragments (Figure 16B). In addition, the distribution of cells in the cell cycle phases indicated an increase in the G0/G1 phase in both treated groups. The group treated with Coenzyme Q10 (5.8 μM) + 2-AEH2P (22.8 mM) showed the most significant reduction in the G2/M phase and an increase in the G0/G1 and S phases compared to the control and treatment with Coenzyme Q10 alone (Figure 16B). There was also no observed reduction in mitochondrial electric potential for normal L929 fibroblasts for any of the treatments to which they were subjected (Figure 17). However, EAT tumor cells treated in combination indicated significant and greater reductions compared to the control and isolated treatments with Coenzyme Q10 (5.5 μM) and 2-AEH2P (45.6 mM) (Figure 17). According to these results, the expression of Bcl2 decreased, and the release of Cytochrome c increased compared to the control and the group treated only with Coenzyme Q10 (5.5 μM) (Figure 18). An increase in TNF-α, Caspase 3, and P53 was also observed in the combined treatments (Figure 18). In accordance with the data, the combination analysis of Coenzyme Q10 + 2-AEH2P showed a score of −17.50 for normal L929 fibroblasts (Figure 19A) and 0.13 for EAT tumor cells (Figure 19B), indicating an antagonistic effect for normal L929 cells and an additive effect for EAT tumor cells under the same treatment conditions.

### 3.5. Analysis of the Compound 2-AEH2P Associated with the Chemotherapeutic Paclitaxel in Murine Fibroblast Cells (L929) and Ehrlich Ascitic Tumor (EAT) Cells

**Figure 20:**
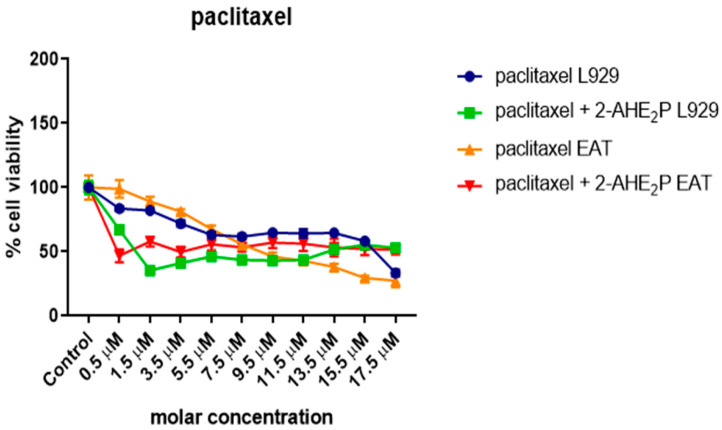
Determination of cytotoxicity in normal murine fibroblasts (L929) and Ehrlich ascitic tumor (EAT) cells using the colorimetric MTT method treated with paclitaxel (serial dilution) and paclitaxel (serial dilution) + 2-AEH2P (22.8 mM). Values are expressed as mean ± SD from three independent experiments. Statistical differences were obtained using the non-parametric ANOVA variance test and Tukey–Kramer multiple comparisons test.

**Figure 21:**
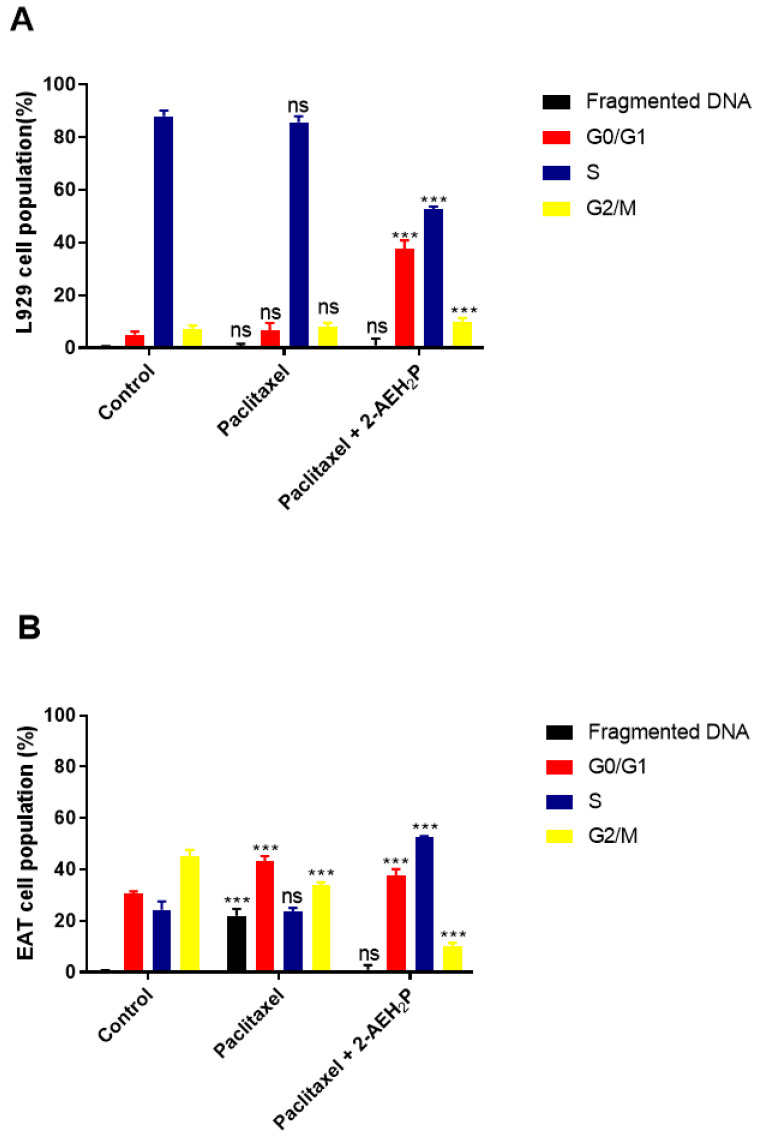
Analysis of cell cycle phases. (**A**) Normal murine fibroblasts (L929); (**B**) Ehrlich ascitic tumor (EAT) cells. Cells were treated with paclitaxel (8.7 μM) and paclitaxel (6.2 μM) + 2-AEH2P (22.8 mM) for a period of 24 h. Representative histograms of cell distribution in cell cycle phases and fragmented DNA, and bar graph showing correlation of cell cycle effect expressed as mean ± SD from three independent experiments. Levels of significance, ns = not significant, *** *p* < 0.001.

**Figure 22:**
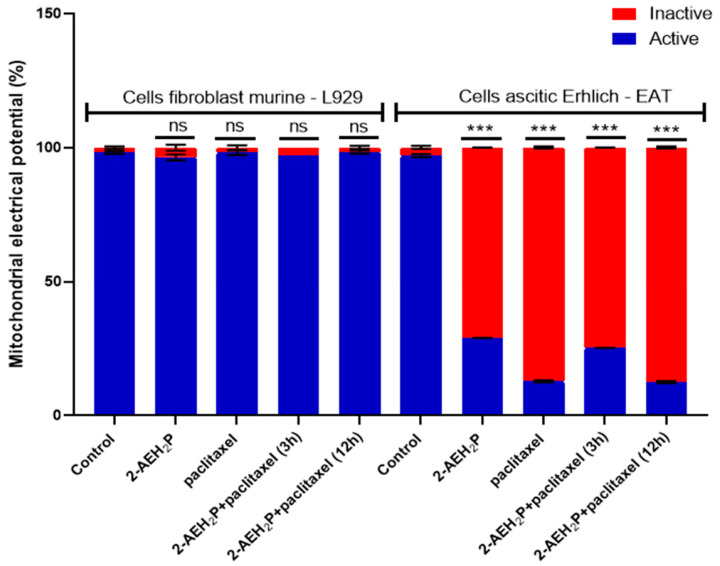
Mitochondrial membrane potential. Normal murine fibroblasts (L929) and Ehrlich ascitic tumor (EAT) cells were treated with 2-AEH2P (45.6 mM), paclitaxel (8.7 μM), and paclitaxel (6.2 μM) + 2-AEH2P (22.8 mM) for a period of 24 h. Bar graph showing the correlation of treatment effect on mitochondria expressed as mean ± SD from three independent experiments. Levels of significance, ns = not significant, *** *p* < 0.001.

**Figure 23:**
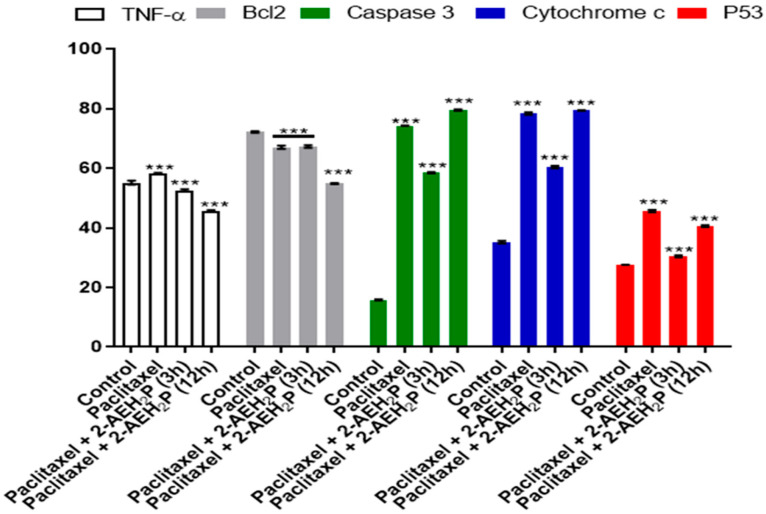
Graph of the expression of markers TNF-α/DR-4, Bcl2, Caspase 3, Cytochrome c, and P53 in Ehrlich ascitic tumor cells treated with paclitaxel (8.7 μM) and paclitaxel (6.2 μM) + 2-AEH2P (22.8 mM). Quantification by flow cytometry, 24 h after treatments. Values are expressed as mean ± SD from three independent experiments. Statistical differences were obtained using the non-parametric ANOVA variance test and Tukey–Kramer multiple comparisons test. Levels of significance, *** *p* < 0.001.

**Figure 24:**
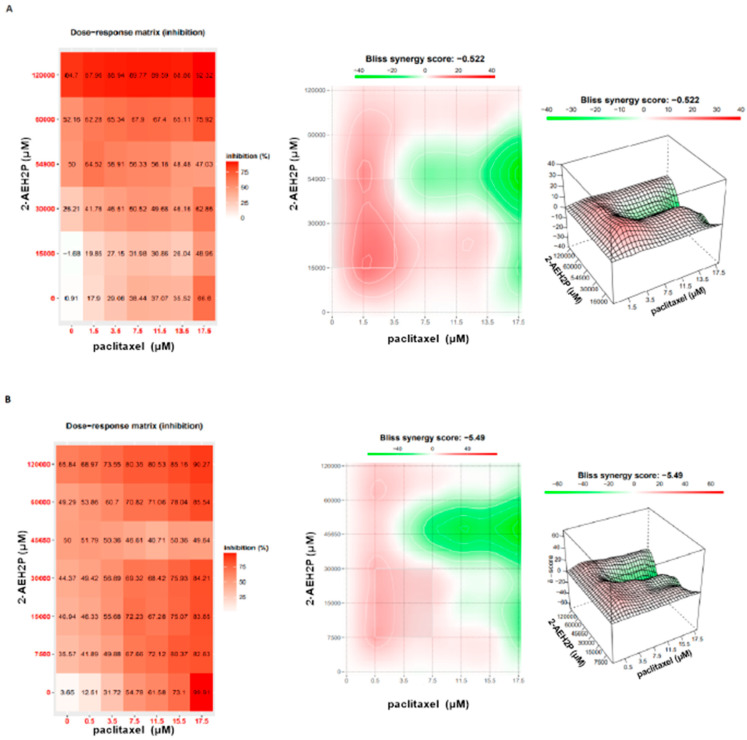
Analysis of the degree of pharmacological interactions. (**A**) Normal murine fibroblast cells (L929); (**B**) Ehrlich ascitic tumor (EAT) cells. Treated with varying concentrations of paclitaxel, 2-AEH2P, and paclitaxel + 2-AEH2P for 24 h. The results of cell viability were subjected to analysis using the Bliss independence model and SynergyFinder. The Bliss independence model logarithmically calculated the expected combinatorial effect based on the probability of independent events caused by the combination of drugs.

Cytotoxicity analysis (Figure 20) for normal L929 fibroblast cells showed an IC50% of 20.7 μM for the paclitaxel-treated group. However, for the associated group, after 3 h of paclitaxel treatment, when 2-AEH2P was added, the IC50% significantly decreased to 1.4 μM. Under the same conditions, the IC50% for the Ehrlich ascitic tumor (EAT) cells was 6.2 μM for the associated group, while cells treated only with paclitaxel had an IC50% of 8.7 μM. Similarly, in EAT tumor cells, when evaluating the cell cycle phase distribution profile (Figure 21), it was noted that the combination of paclitaxel (6.2 μM) + 2-AEH2P (22.8 mM) showed no modifications in the proportions of cells with fragmented DNA. This result differs from the group treated only with paclitaxel (Figure 21B) and from previous paclitaxel + 2-AEH2P association results. In normal murine L929 fibroblasts, the paclitaxel-treated group showed no significant differences in cell cycle phases (Figure 21A). However, the association modulated the distribution of cell cycle phases, although no fragmented DNA was observed in the treatments: paclitaxel (8.7 μM) and paclitaxel (6.2 μM) + 2-AEH2P (22.8 mM) for normal L929 fibroblasts. This result is related to the chemotherapy action time on the cell, considering the paclitaxel action time in the cell cycle. When evaluating mitochondrial membrane potential and marker expression, tests were conducted on L929 fibroblasts and EAT tumor cells after 3 or 12 h between paclitaxel (6.2 μM) and 2-AEH2P (22.8 mM) administration. For the evaluation of mitochondrial membrane potential (Figure 22), in normal murine L929 fibroblasts, no changes in membrane potential were observed under any conditions. In contrast, EAT tumor cells showed a decrease in mitochondrial membrane potential in all treatments (Figure 22). It is worth noting that, under association conditions at 3 h, the membrane potential was 26.1 ± 0.23%, compared to the treatment with paclitaxel alone, which showed 13.4 ± 0.56%. The association after 12 h was 12.6 ± 0.48%, thus showing the lowest observed membrane potential among all treatments and associations. Regarding marker expression, EAT tumor cells treated in association showed a significant decrease in the expression of TNF-α/DR-4 and Bcl2, and an increase in the expression of Caspase 3, release of Cytochrome c, and P53 (Figure 23). Only the paclitaxel-treated group (8.7 μM) showed an increase in TNF-α expression. The pharmacological analysis of the paclitaxel (6.2 μM) + 2-AEH2P (22.8 mM) combination showed an additive effect for both cell lines, with a synergistic score of −0.52 (Figure 24A) for normal L929 fibroblasts and −5.49 (Figure 24B) for tumor cells.

## 4. Discussion

Although the mechanism of action of the monophosphoester 2AEH2P is still under investigation, a set of studies evaluating its action in cancer indicates that the compound exhibits selective specificity for tumor cell lines. Recently, in triple-negative breast cancer cells MDA-MB-321, its activity was related to the decrease in CD44, CD24, Bcl2, release of Cytochrome c to the cytoplasm, and mitochondrial reduction [49]. In MCF-7 breast cancer cells, deficient in caspase 3 expression, 2-AEH2P was able to induce its activation, detected by the cleavage of a specific caspase-3 substrate, Ac-YVAD-AMC [25]. Similarly, when associated with liposomal formulation in K-562 and K-562 Lucena (MDR+) tumor cells, a reduction in mitochondrial electrical potential, Bcl-2, increased expression of the pro-apoptotic proteins BAD, BAX, and phosphorylated caspase 3 and 8, as well as Cytochrome c release in the cytoplasm were observed^27^. In accordance, our data also indicated that in Ehrlich ascitic tumor (EAT) cells, 2-AEH2P altered the distribution of cells in cell cycle phases and the expression of pro- and anti-apoptotic proteins, both alone and when combined with other drugs. It is possible that the pro-apoptotic effects of 2-AEH2P are related to mitochondrial destabilization, as tumor cells require more energy produced by mitochondria to maintain their proliferation rate and rapid cell division. The lack of growth inhibition in normal cells may be related to their stability in mitochondrial activities. Pro-apoptotic stress induced by activated BAX and BAK alters the permeabilization of the mitochondrial outer membrane (MOMP), allowing the release of soluble proteins from the intermembrane space, including Cytochrome c [50]. In our results, caspase-8 expression, related to the activation of the extrinsic apoptotic pathway [50], was not evaluated; however, our data indicate the action of 2-AEH2P promoting apoptosis through the intrinsic pathway. It is important to note that the significant role of mitochondrial metabolism in tumorigenesis could be potentially explored as a strategy for cancer therapy [51]. In the tumor microenvironment, tumor cells and immunosuppressive myeloid cells show an abundance of cholesterol [52]. Therefore, statins, competitive inhibitors of HMG-CoA reductase (HMGCR), have attracted much attention for their anticancer properties [53], including Simvastatin. However, evidence of its effectiveness is inconsistent [52]. Considering the pro-tumoral characteristics of cholesterol and its ability to stiffen the cell membrane, increasing multidrug resistance (MDR) [54], the association between Simvastatin + 2-AEH2P is likely to allow Simvastatin, by inhibiting cholesterol synthesis, to increase the permeability of the cell membrane, allowing 2-AEH2P to potentiate its biological activity at a lower concentration. In another study, it was shown that decreasing the cholesterol synthesis pathway in HCC1143 breast cancer cells reduced their ability to form tumor spheres and invasion [9]. The results obtained through the evaluation of the pharmacological effects of marker expression between the 2-AEH2P + GM-CSF association enable new clinical protocols in search of therapeutic strategies targeting mitochondria and cancer immunotherapy. Mitochondria, responsible for numerous physiological and metabolic processes [55], are related to a pro-tumoral effect when dysfunctional. Several studies show their role in the immunoregulatory function of immune cells, as well as their impact on the tumor microenvironment [56,57,58,59]. The regulation of macrophages, NK cells, and T cells contributes to immune escape by reducing immunogenic antigens and decreasing the cytotoxic activity of T cells [59]. The consumption of Coenzyme Q10, as established by the FDA, is widely available only as a dietary supplement [23]. Several studies bring contradictions regarding its action in the tumor microenvironment. The variable success in the results of randomized clinical trials supplementing Coenzyme Q10 may be associated with a series of unresolved issues related to its metabolism [60]. In EAT cells, the combination of Coenzyme Q10 with the compound 2-AEH2P showed an additive pharmacological effect. Both alone and in combination with 2-AEH2P, an increase in fragmented DNA was observed. However, the level of tumor cells in the G0/G1 phase of the cell cycle was significantly higher compared to the control. In addition, compared to the control, this treatment resulted in a decrease in cells in the G2/M phase, decreased expression of Bcl2, mitochondrial electrical potential, release of Cytochrome c, and an increase in the expression of pro-apoptotic proteins. In a 3-year study with stage I and II melanoma patients, a significant decrease in recurrence rates and adverse effects was observed in those who received Coenzyme Q10 supplementation, along with low doses of interferon α-2 [61]. Despite the small number of patients and the short duration, the evaluation of a potential approach with Coenzyme Q10 supplementation in cancer patients highlighted overall improvement in inflammatory, oxidative, and specific biochemical markers [61,62]. The lack of homogeneity in results stimulates further investigation into its synergistic effect associated with 2-AEH2P, especially considering the presented data. The combination of paclitaxel + 2-AEH2P, investigated by our group in triple-negative breast cancer 4T1 cells, increased the antiproliferative potential, exhibiting morphological changes such as cytoplasmic retraction and regression of the cytoskeleton [63]. It also showed additive pharmacological effects that may favor antitumor therapy and attenuate systemic toxicity. It is worth noting that the presence of high levels of TNF-α expressed in tumor cells may be related to the induction of an inflammatory response. A recent study described a mitochondrial mechanism, inflammation, and mechanisms inducing cell death, where inflammation plays a fundamental role, activating pro-inflammatory pathways during cell death after permeabilization of the mitochondrial outer membrane (MOMP) [64]. In addition, it was suggested that caspases play a regulatory anti-inflammatory role shortly after reaching the fundamental compromise point, initiating apoptosis [64]. When clinically evaluated in phase I/II in dogs with tumors, 2-AEH2P was safe at all escalated doses up to 150 mg/kg for 8 weeks [65]. The intravenous administration did not show mortality during the study period, acute toxicity, hemolysis, anemia, or changes in liver and kidney functions. Further studies are needed to elucidate the mechanism of action of the compounds when combined. However, the data presented in this study effectively demonstrate the involvement of the compounds in inducing cell death, and their combinatorial action showed pharmacological synergy, increasing the action potential of 2-AEH2P in all associated treatments.

## 5. Conclusions

The toxicity values (IC50%) for EAT tumor cells decreased when subjected to combination treatments, with the exception of the combination treatment with the chemotherapy paclitaxel + 2-AEH2P, despite showing an antagonistic effect in the synergistic evaluation. When tumor cells were treated with chemotherapy for 12 h before the administration of 2-AEH2P, we observed the greatest reduction in mitochondrial electrical potential among all tested combinations in tumor cells; no effects were observed for normal cells. The compounds associated with 2-AEH2P exhibited a significant synergistic effect, with modulation of marker expression being observed. The isolated treatment of 2-AEH2P for normal cells showed a proliferative effect at concentrations below 15 mM; however, this mechanism was not evaluated in this study. Pharmacological analysis indicated that tumor cells treated with GM-CSF + 2-AEH2P exhibited a synergistic effect, while groups of tumor cells treated with paclitaxel, Coenzyme Q10, and Simvastatin associated with 2-AEH2P showed additive effects, demonstrating adjunct therapeutic effects in controlling tumor cell proliferation.

## Figures and Tables

**Table 1:** Concentrations used for the analysis of cell cycle phases and analysis of mitochondrial membrane potential in normal murine fibroblast cells (L929) and Ehrlich ascitic tumor (EAT) cells. Concentrations obtained by analyzing cytotoxic activity using the MTT colorimetric method.

Treatment	Drug Concentration
2-AEH2P	45.6 mM
Simvastatin	9.6 μM
GM-CSF	144 nM
Coenzyme Q10	5.5 μM
Paclitaxel	8.7 μM
Simvastatin + 2-AEH2P	10.1 μM + 22.8 mM
GM-CSF + 2-AEH2P	16 nM + 22.8 mM
Coenzyme Q10 + 2-AEH2P	5.8 μM + 22.8 mM
Paclitaxel + 2-AEH2P	6.2 μM + 22.8 mM

## Data Availability

The data presented in this study are available in this article.
